# Delayed spontaneous rupture of cavernous segment of the internal carotid artery following dual ophthalmic segment aneurysms treatment with pipeline embolization device: A case report

**DOI:** 10.1097/MD.0000000000018420

**Published:** 2019-12-27

**Authors:** Wang Ting, Seidu A. Richard, Zhang Changwei, Wang Chaohua, Xie Xiaodong

**Affiliations:** a Department of Neurosurgery, West China Hospital, Sichuan University, Chengdu, P.R. China; b Department of Medicine, Princefield University, Ghana West Africa.

**Keywords:** CCF, dual, ICA, ophthalmic segment, PLED

## Abstract

**Rationale:**

The incidence of double aneurysms on the ophthalmic segment of the internal carotid artery (ICA) is very rare. Nevertheless, delayed rupture of a parent artery instead of the aneurysmal sac following pipeline embolization device (PLED) is unusual.

**Patient concerns:**

We present a 72-year-old female who was admitted at our facility with gradual onset of nonspecific visual changes.

**Diagnosis:**

Conventional angiography revealed 2 aneurysms located at the ophthalmic segment of the left ICA.

**Interventions:**

Both aneurysms were successfully treated with PLED.

**Outcomes:**

Two months after discharge, the patient was rushed into the emergency with bilateral conjunctival congestion. Computed tomography revealed intracranial hemorrhage at left temporal lobe while digital subtraction angiography established a left direct carotid cavernous fistula. We utilized stent (Solitaire 6∗30) assisted coils to occlude the fistula. The patient is well and go about her normal duties.

**Lessions:**

Manipulation of the tortuous parent artery resulted in a focal traumatic weakness in the artery and subsequently a delay tear. We are of the view that, endovascular surgeons should be on the lookout for this complication following flow deviation treatment modalities.

## 1 Introduction

The occurrence of dual aneurysms on the ophthalmic segment of the internal carotid artery (ICA) is very rare. Rupture of the parent artery instead of the aneurysmal sac following Pipeline embolization device (PLED) is very unusual.^[1]^ In most cases reported, is usually the aneurysmal sac that often ruptures and not a healthy parent artery.[^2^,^3^,^4^,^5^,^6^] PLEDs works by channeling blood flow away from the aneurysm into the parent vessel.^[7]^ This often result in stasis of blood flow within the aneurysm, inflammation and/or thrombosis and, finally, total elimination of the aneurysm from circulation through endothelial proliferation on the device.^[7]^ We report a case of delayed spontaneous rupture of cavernous segment of the ICA following dual ophthalmic segment aneurysms (OSA) treatment with PLED.

## 2 Case report

**Figure 1:**
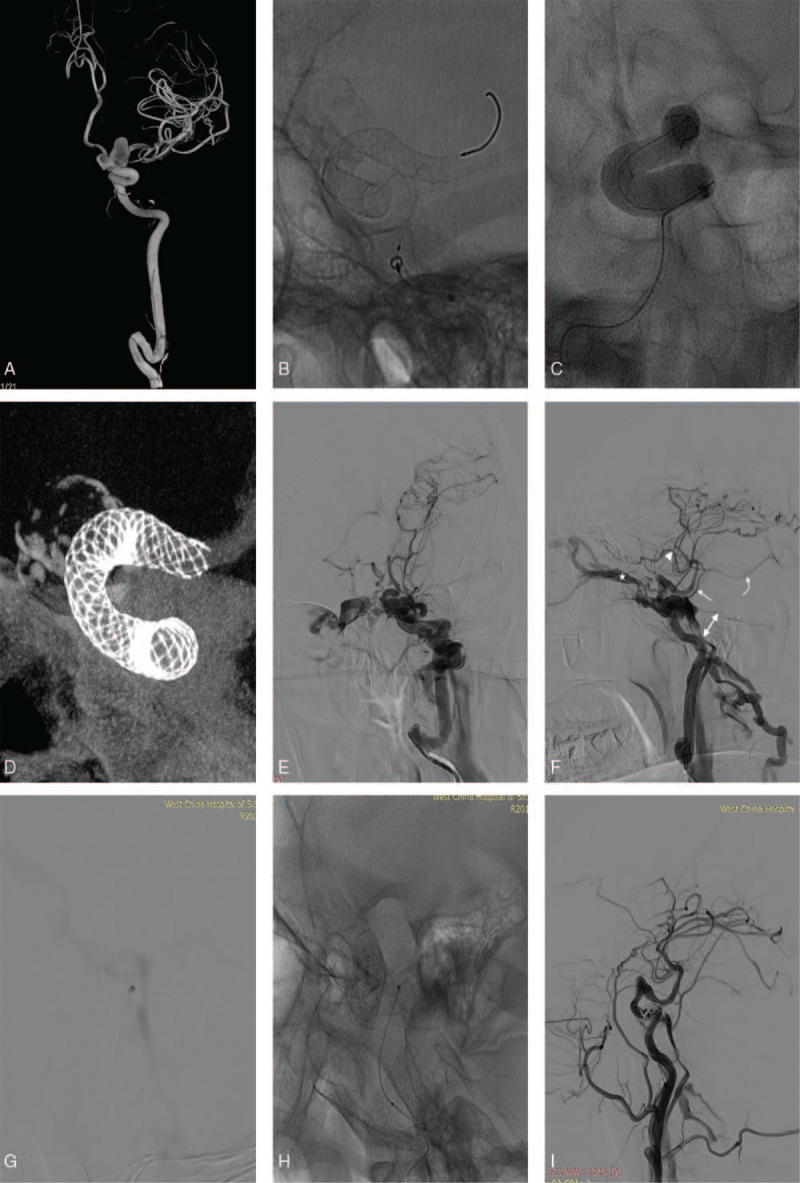
(A) Is a conventional angiography showing 2 aneurysms located at ophthalmic segment of the left ICA. (B) Is intraoperative DSA showing inability of the PLED to open well at the anterior bend of the siphon because of the tortuosity of the parent artery. (C) Is an intraoperative showing straightening of tortuous of the parent artery with HyperGlide5∗20 (Medtronic) balloon. (D) Is a postoperative DSA showing perfect implantation of the PLED across both aneurysmal necks. (E and F) Are DSA images showing a left direct CCF with drainage from the bilateral superior ophthalmic veins, left superior and inferior petrosal sinuses, superficial middle cerebral vein, anterior cerebral vein, and basal vein. (G and H) Are intraoperative DSA images showing the rupture point proximal to the PLED and not the aneurysmal wall or sac. (I) Is a postoperative DSA, showing no draining veins except the inferior petrosal sinus. DSA = digital subtraction angiography, ICA = internal carotid artery, PLED = pipeline embolization device.

A 72-year-old female was admitted at our facility with gradual onset of nonspecific visual changes. General physical examination was unremarkable. Fundoscopic examination did not yield much. Conventional angiography revealed 2 aneurysms at ophthalmic segment of the left ICA (Fig. 1A). We treated both aneurysms with PLED (ev3/Covidien, Irvine, CA) without coils under general anesthesia. Intraoperatively, the PLED did not open well at the anterior curve of the siphon (Fig. 1B) because the parent artery was tortuous, so we use HyperGlide5∗20 (Medtronic, Neurovascular, Irvine, CA) balloon to straighten the tortuous parent artery (Fig. 1C). We achieved perfect implantation of the PLED across both aneurysmal necks’ after straightening the tortuous parent artery (Fig. 1D). The patient was put on Aspirin 100 mg once daily and Clopidogrel 75 mg once daily after the procedure. She was discharged home on postoperative day 5 after repeated conventional angiography detected no complications. The aneurysm was totally occluded before discharge.

Two-month after the operation, the patient was rushed into the emergency room. Physical examination revealed bilateral conjunctival congestion while emergency Computed tomography revealed intracranial hemorrhage at left temporal lobe. Digital subtraction angiography (DSA) established a left direct carotid cavernous fistula (CCF) with drainage via the bilateral superior ophthalmic veins, left superior and inferior petrosal sinuses, superficial middle cerebral vein, anterior cerebral vein and basal vein (Fig. 1E and F). Intraoperatively, we advanced enchelon-10 (Medtronic) into the fistula. We detected that the rupture point was proximal to the PLED (Fig. 1G and H) and not the aneurysmal wall or sac. We used stent (Solitaire 6∗30) assisted coils to occlude the fistula. On post procedure DSA, we observed that, the all the draining veins were eliminated except for the inferior petrosal sinus (Fig. 1I). We discharged the patient 2 weeks after the procedure. The intracranial hemorrhage was absorbed before discharge. Two years follow-ups revealed that the patient is well with no neurological deficits and no recurrence of the fistula.

## 3 Discussion

OSA of the ICA constitutes about 5% to 11% of all intracranial aneurysms.^[8]^ They often advance from smaller sizes into large or giant over a period of time. In 50% of cases, aneurysm on this segment occurs concurrently with other intercranial aneurysms. Bilateral incidence account for about 7% of patients with OSA.[^8^,^9^,^10^] Nevertheless, dual occurrence of aneurysms on the same ophthalmic artery is very rare. This; therefore, makes our case very interesting. The management of OSAs is very problematic to neurosurgeons because of poor outcomes.[^8^,^10^]

Rehman et al reported the first case of hemorrhage from vessel rupture instead of the aneurysmal sac following treatment of basilar artery aneurysm with PLAD.^[1]^ Their patient died 3 days after the procedure and the diagnosis of artery rupture instead of the aneurysmal sac was established on autopsy.^[1]^ We also observed delayed spontaneous rupture of cavernous segment of the ICA following dual OSA treatment with PLED. Nevertheless, our patient did not die. We also did not observe this complication immediately after the procedure.

Rehman et al indicated that, the aneurysm sac in their case was absolutely intact but the basilar artery developed a focal rupture in an area that was covered by the PLED.^[1]^ They did not believe the basilar artery rupture in their case was iatrogenic because they did not observe hemorrhage immediately after the procedure.^[1]^ We observed hemorrhage 2 months after treating the patient with the PLAD. This mean that the rupture of the cavernous segment of the ICA in our case was a delayed spontaneous event instead of an iatrogenic event. Furthermore, we observe this complication during postoperative evaluation and our patient is still alive and well. We used DSA to identify the rupture point or the fistula on the ICA. The drainage the CCF was via bilateral superior ophthalmic veins, left superior and inferior petrosal sinuses, superficial middle cerebral vein, anterior cerebral vein, and basal vein.

Delayed hemorrhage following PLED implantation in cerebral aneurysms has been reported.[^2^,^3^,^4^,^5^,^6^] The most potential mechanisms via aneurysm rupture leading to hemorrhage is aneurysm wall inflammation and/or thrombosis.[^1^,^4^,^11^] Aneurysmal rupture is not particular to PLED alone. Similar occurrences have also been reported following implantation of the Silk flow diverters.[^2^,^12^] In most cases, the aneurysm rupture is usually delayed although subacute cases have been reported.^[2]^

Rehman et al noted that, the rupture point or fistula was adjacent to an atheromatous plaque.^[1]^ We did not observe an such predisposing factors in our patient. Their findings were established during autopsy examination of the patient. They assumed that, vessel “stretching” and stress at the weak point where the device also tapers into the smaller left posterior communicating artery could have caused rupture of the vessel.^[1]^ We are also of the view that, radial force exerted by the PLED coupled the flow dynamics in vessel led to rupture of the vessel in our case.

They further speculated that unrecognized intraprocedural intimal vessel injury that consequently dissected through the vessel wall could be the cause of rupture.^[1]^ Nevertheless, we did not think the rupture in patient was of iatrogenic origin although we cannot rule out the fact the dual nature OSA coupled with a tortuous parent artery led to several technical manipulation which could have resulted in a focal traumatic weakness in artery resulting in a delay tear.

## 4 Conclusion

Dual occurrence of OSA is very rare. Rupture of the parent artery instead of the aneurysmal sac following PLED implantation is very unusual. We are of the view that, endovascular surgeons should be on the lookout for this complication following flow deviation treatment modalities. Manipulation of the tortuous parent artery can result in a focal traumatic weakness in the artery and subsequently a delay tear.

## Author contributions

**Conceptualization:** Wang Ting, Seidu A. Richard, Zhang Changwei, Wang Chaohua, Xiao Dong Xie.

**Data curation:** Wang Ting, Seidu A. Richard, Zhang Changwei, Wang Chaohua, Xiao Dong Xie.

**Formal analysis:** Wang Ting, Seidu A. Richard, Zhang Changwei, Wang Chaohua, Xiao Dong Xie.

**Funding acquisition:** Xiao Dong Xie.

**Methodology:** Seidu A. Richard.

**Resources:** Zhang Changwei.

**Supervision:** Zhang Changwei, Wang Chaohua, Xiao Dong Xie.

**Writing – original draft:** Seidu A. Richard.

**Writing – review and editing:** Wang Ting, Seidu A. Richard, Zhang Changwei, Wang Chaohua, Xiao Dong Xie.
